# Haplotype-resolved genome assembly of *Corydalis yanhusuo*, a traditional Chinese medicine with unusual telomere motif

**DOI:** 10.1093/hr/uhad296

**Published:** 2024-01-02

**Authors:** Dingqiao Xu, Ziqi Ye, Yongji Huang, Kejin Zhu, Hongbo Xu, Jingao Yu, Yimeng Feng, Xinyue Zhao, Long Wang, Hao Xu, Qien Li, Minjian Qin, Yuping Tang, Xingtan Zhang, Yucheng Zhao

**Affiliations:** School of Pharmacy, Shaanxi University of Chinese Medicine, Xi’an 712046, China; Genome Analysis Laboratory of the Ministry of Agriculture, Agricultural Genomics Institute at Shenzhen, Chinese Academy of Agricultural Sciences, Shenzhen 518000, China; Marine and Agricultural Biotechnology Laboratory, College of Geography and Oceanography, Minjiang University, Fuzhou 350108, China; Department of Resources Science of Traditional Chinese Medicines, School of Traditional Chinese Pharmacy, China Pharmaceutical University, Nanjing 210009, China; Shaanxi Innovative Drug Research Center, Shaanxi University of Chinese Medicine, Xi’an 712046, China; Shaanxi Innovative Drug Research Center, Shaanxi University of Chinese Medicine, Xi’an 712046, China; Department of Resources Science of Traditional Chinese Medicines, School of Traditional Chinese Pharmacy, China Pharmaceutical University, Nanjing 210009, China; Department of Resources Science of Traditional Chinese Medicines, School of Traditional Chinese Pharmacy, China Pharmaceutical University, Nanjing 210009, China; Department of Resources Science of Traditional Chinese Medicines, School of Traditional Chinese Pharmacy, China Pharmaceutical University, Nanjing 210009, China; School of Bioscience and Engineering, Shaanxi University of Technology, Hanzhong 723000, China; Tibetan Medicine Research Center of Qinghai University, Tibetan Medical College of Qinghai University, Xining 810016，China; Department of Resources Science of Traditional Chinese Medicines, School of Traditional Chinese Pharmacy, China Pharmaceutical University, Nanjing 210009, China; School of Pharmacy, Shaanxi University of Chinese Medicine, Xi’an 712046, China; Genome Analysis Laboratory of the Ministry of Agriculture, Agricultural Genomics Institute at Shenzhen, Chinese Academy of Agricultural Sciences, Shenzhen 518000, China; Department of Resources Science of Traditional Chinese Medicines, School of Traditional Chinese Pharmacy, China Pharmaceutical University, Nanjing 210009, China

Dear Editor,

*Corydalis yanhusuo* (‘Yuan-hu’ or ‘Yan-hu-suo’ in Chinese) belongs to the genus *Corydalis* of the Papaveraceae family. It is widely used in traditional Chinese medicine and honored as one of the eight genuine Chinese medicines in Zhejiang. It is well known for its medical role in pain relief, activating blood and resolving stasis, reducing drug addiction, treating cardiovascular diseases, and so on [1, 2]. Despite researchers having proved that benzylisoquinoline alkaloids (BIAs), such as tetrahydropalmatine, corydalis, and protopine, are the main active ingredients of *C. yanhusuo*, little is known about its genetics [1, 3]. Herein, we report a chromosome level and haplotype-resolved genome assembly of *C. yanhusuo* to provide a genetic resource for molecular breeding and biosynthetic pathway parsing of BIAs.

The genome size of *C. yanhusuo* was firstly estimated to be 1.76 Gb using *k*-mer analyses. The *k*-mer frequency–depth distribution did not confirm to the Poisson distribution, and showed high heterozygosity and duplication ratios, which were 1.69 and 52.01%, respectively (Fig. 1A). The above results implied that the *C. yanhusuo* genome is extremely complex. To ascertain chromosome number and ploidy, we conducted fluorescence *in situ* hybridization (FISH) mapping on the metaphase chromosomes of root tip meristem cells using 5S rDNA probes. Four loci were generated on 32 metaphase chromosomes, suggesting that *C. yanhusuo* is a tetraploid with 32 chromosomes (2*n* = 4*x* = 32, Fig. 1B).

The contigs were initially assembled with hifiasm software using HiFi PacBio subreads, yielding a contig-level sequence of 2.18 Gb for the *C. yanhusuo* genome (Fig. 1C). Next, we applied the ALLHiC pipeline to construct a chromosome-scale assembly using a polyploid model. All contigs were first partitioned into eight homologous groups following the allele table, and then ALLHiC_partition was applied to separate each homologous group into four groups. We also improved the genome assembly at the chromosomal level using RagTag to determine the ordering and orientation of the contigs in each group (Fig. 1C). The heat map clearly demonstrated the clustering of the 32 pseudochromosomes into eight homoeologous groups (Fig. 1D). In addition to the Hi-C interaction heat map, the satisfactory collinearity observed in the genome comparison between the entire *C. yanhusuo* genome and the diploid *Corydalis hendersonii* (unpublished genome) (Fig. 1E) further supports the high-quality genome assembly of the former.

**Figure 1:**
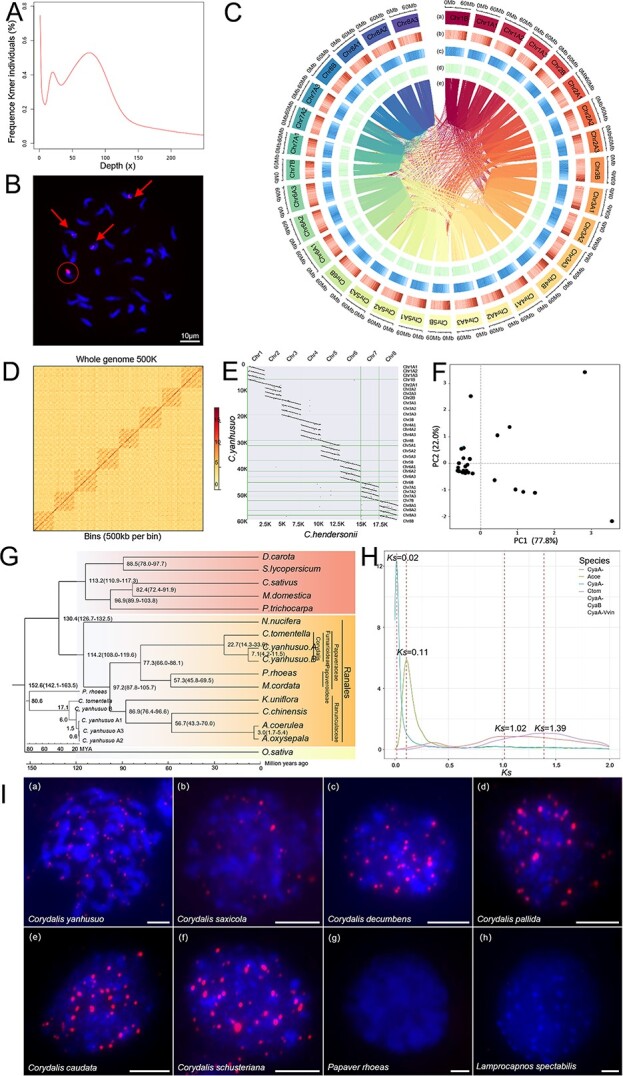
**A** *K*-mer analysis of *C. yanhusuo* genome size. **B***Corydalis yanhusuo* cells present an auto-allotetraploid karyotype with 16 pairs of chromosomes. The arrows represent three similar haplotypes (we designate them as type A) and the circle represents one special haplotype (type B). **C** Genomic features of 32 pseudochromosomes. Notations (a)–(e) refer to chromosomes, gene density, repeat sequence density, GC content, and synteny between pseudochromosomes, respectively. **D** Hi-C heat map for 32 assembled pseudochromosomes. Each homoeologous group contains four pseudochromosomes. **E** Collinearity comparison of *C. yanhusuo* with *C. hendersonii.***F** Principal component analysis of the differential 15-mers (represented by the black spot on the left and the black spot on the right). **G** Phylogenetic tree of 14 plants and the A/B subgenomes of *C. yanhusuo*. The phylogenetic tree of different subgenomes of *C. yanhusuo* using *P. rhoeas* and *C. tomentella* as ancestral chromosomes is also shown in **G**. **H***K*_s_ distribution. The abbreviations CyaA, CyaB, Acoe, Ctom, and Vvin represent *C. yanhusuo* subgenome A, *C. yanhusuo* subgenome B, *Aquilegia coerulea*, *C. tomentella*, and *Vitis vinifera*, respectively. **I** Telomere detection of species from Papaveraceae using FISH.

To further confirm the chromosome phasing results, we re-examined the chromosome ploidy of *C. yanhusuo*. As indicated in the FISH results, there are three similar haplotypes (marked with arrows in Fig. 1B; we designated them as type A) and one special haplotype (marked with a circle in Fig. 1B, designated type B). Principal component analysis and the cluster heat map of the differential 15-mers supported this result (Fig. 1F), demonstrating that SubPhaser can nearly phase the subgenomes in a 3:1 proportion with two different karyotypes (represented by the black spot on the left and the black spot on the right in Fig. 1F). An intriguing result from the Smudgeplot analysis suggested that the genome structure of *C. yanhusuo* may be AB or another structure. Consequently, we proposed that *C. yanhusuo* may be an auto-allopolyploid (a special tetraploid with three homologous chromosomes and one non-homologous chromosome) with a special AAAB karyotype. According to this hypothesis, the 32 pseudochromosomes were split into four haplotypes (Fig. 1C). The N50 values for each haplotype of the tetraploid *C. yanhusuo* genome were 61.25, 60.71, 60.46, and 62.22 Mb. A total of 126 230 protein-coding genes were predicted, of which 93.31% (11 7791) were functionally annotated in the KEGG, Nr, UniProt, and other databases. The overall genome assembly quality was assessed according to the long terminal repeat (LTR) retrotransposons and BUSCO; 64.35% of the *C. yanhusuo* genome was annotated as transposable elements (TEs) and gene completeness was 97%.

A phylogenetic tree was constructed using 145 single-copy genes, which are present in ~90% of the species (Fig. 1G). Subgenomes A and B of *C. yanhusuo* are estimated to have diverged ~7.1 million years ago (MYA). The subgenomes A2 and A3 of *C. yanhusuo* were split from A1 ~1.5 MYA (Fig. 1G). This divergence arose after the divergence with *Corydalis tomentella*, which occurred around 22.7 MYA. Comparisons with Papaveroideae species such as *Papaver rhoeas* and *Macleaya cordata* revealed that the two subfamilies diverged around 77.3 MYA. Papaveraceae are estimated to have split from Ranunculaceae ~97.2 MYA. This is largely in accordance with a previous report on *C. tomentella* [4]. Furthermore, we observed peaks in the *K*_s_ distributions for subgenomes A and B of *C. yanhusuo* at 0.02, while the *K*_s_ peak for orthologs between *C. tomentella* and *C. yanhusuo* is 0.11, providing further evidence of the divergence between the common ancestor of *C. yanhusuo* subgenomes A and B and *C. tomentella* (Fig. 1H). The proximity of these values to the previously reported *K*_s_ peak (*K*_s_ ≈ 1.04) in *C. tomentella* suggests the possibility of a shared whole-genome duplication event during their evolutionary history [4].

To further evaluate the quality of the assembled *C. yanhusuo* genome, we examined the telomere sequences of each chromosome. Intriguingly, none of the chromosomes have the typical angiosperm telomere sequences (TTTAGGG)*_n_*. Instead, we identified a unique repeat motif (TTTCGG)*_n_* in *C. yanhusuo*. To validate the distribution of the repeat (TTTCGG)*_n_*, we performed FISH analysis with repeat (TTTCGG)*_n_* probes on the metaphase chromosomes of root tip meristem cells. Unambiguous FISH signals were detected on the ends of metaphase chromosomes. To investigate whether this unusual telomere motif is ubiquitous in *Corydalis* genomes, the presence of the repeat (TTTCGG)*_n_* was assessed in five other representative plants of this genus with different evolutionary taxonomy [5]. As illustrated in Fig. 1a–f, FISH signals of the (TTTCGG)*_n_* telomere were detected in interphase cells of all selected *Corydalis* species, while no signals were produced in two Papaveraceae species, *P. rhoeas* and *Papaver nudicaule* (Fig. 1g and h). This result implies that the special telomere sequence is common within the genus *Corydalis.*

In this study, we proposed *C. yanhusuo* have a special AAAB karyotype, and therefore assembled a high-quality haplotype-resolved genome of *C. yanhusuo*. At the same time, we detected an unusual (TTTCGG)*_n_* telomere motif that may be highly conserved within the genus *Corydalis*.

## Author contributions

Y.C.Z., X.T.Z., and Y.P.T. conceived and designed the research. D.Q.X., Z.Q.Y., H.B.X., J.G.Y., K.J.Z., and L.W. collected the samples. D.Q.X., Z.Q.Y., and Y.J.H. performed the genome assembly, experimental and data analysis. Y.M.F., X.Y.Z., H.X., L.Q.N., and M.J.Q. also performed the experimental and data analysis. D.Q.X. wrote the manuscript. Y.C.Z., X.T.Z., Z.Q.Y., and Y.J.H. revised the manuscript.

## References

[1] Xu D , Lin H, Tang Y. et al. Integration of full-length transcriptomics and targeted metabolomics to identify benzylisoquinoline alkaloid biosynthetic genes in *Corydalis yanhusuo*. Hortic Res. 2021;8:16 33423040 10.1038/s41438-020-00450-6 PMC7797006

[2] Xia GY , Fang DJ, Wang LY. et al. 13,13a-Seco-protoberberines from the tubers of *Corydalis yanhusuo* and their anti-inflammatory activity. Phytochemistry. 2022;194:113023 34839130 10.1016/j.phytochem.2021.113023

[3] Bu J , Zhang X, Li Q. et al. Catalytic promiscuity of *O*-methyltransferases from *Corydalis yanhusuo* leading to the structural diversity of benzylisoquinoline alkaloids. Hortic Res. 2022;9:uhac152 36168544 10.1093/hr/uhac152 PMC9510826

[4] Xu Z , Li Z, Ren F. et al. The genome of *Corydalis* reveals the evolution of benzylisoquinoline alkaloid biosynthesis in Ranunculales. Plant J. 2022;111:217–30 35476217 10.1111/tpj.15788 PMC7614287

[5] Ren FM , Wang YW, Xu ZC. et al. DNA barcoding of *Corydalis*, the most taxonomically complicated genus of Papaveraceae. Ecol Evol. 2019;9:1934–45 30847083 10.1002/ece3.4886 PMC6392370

